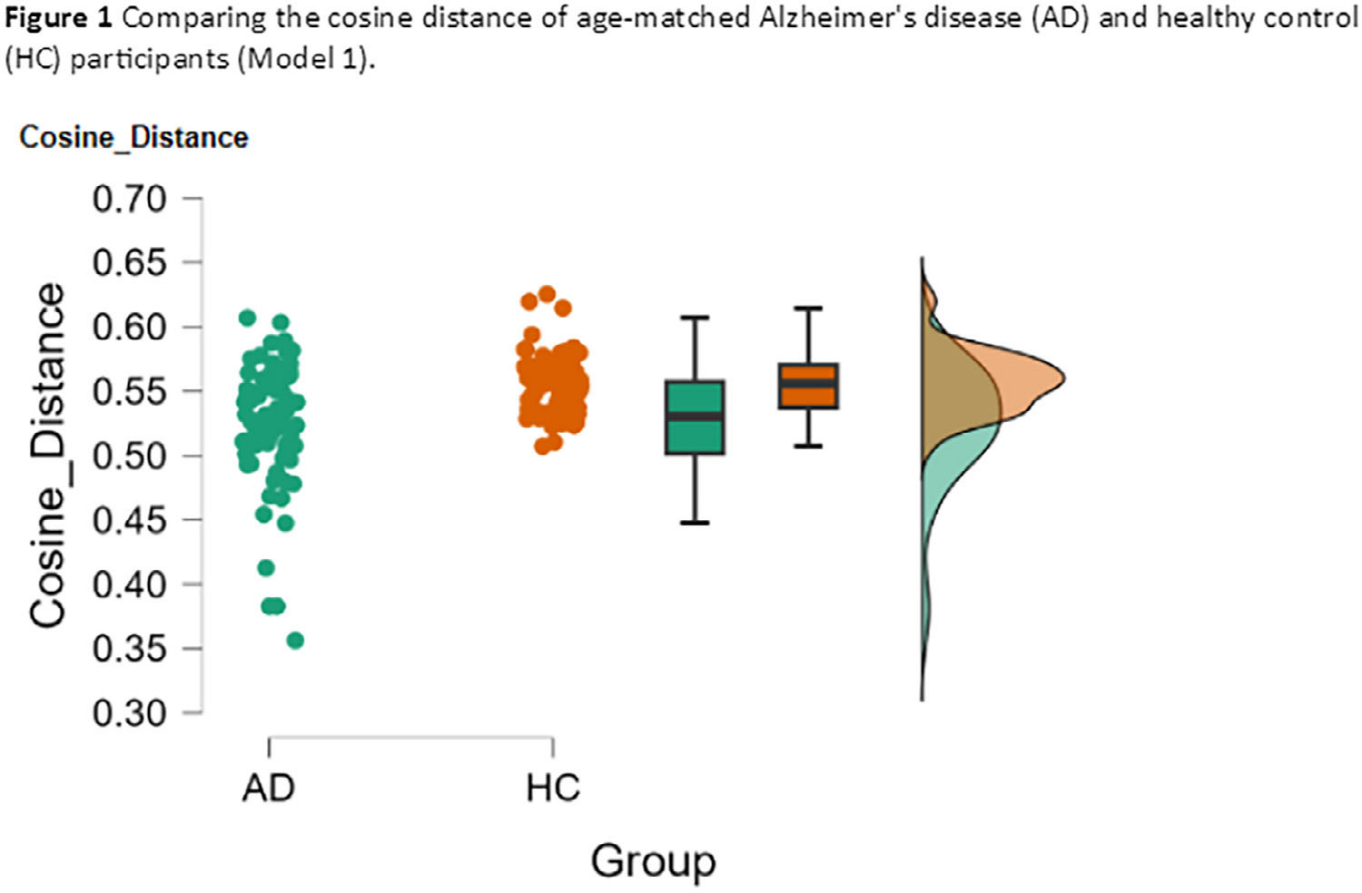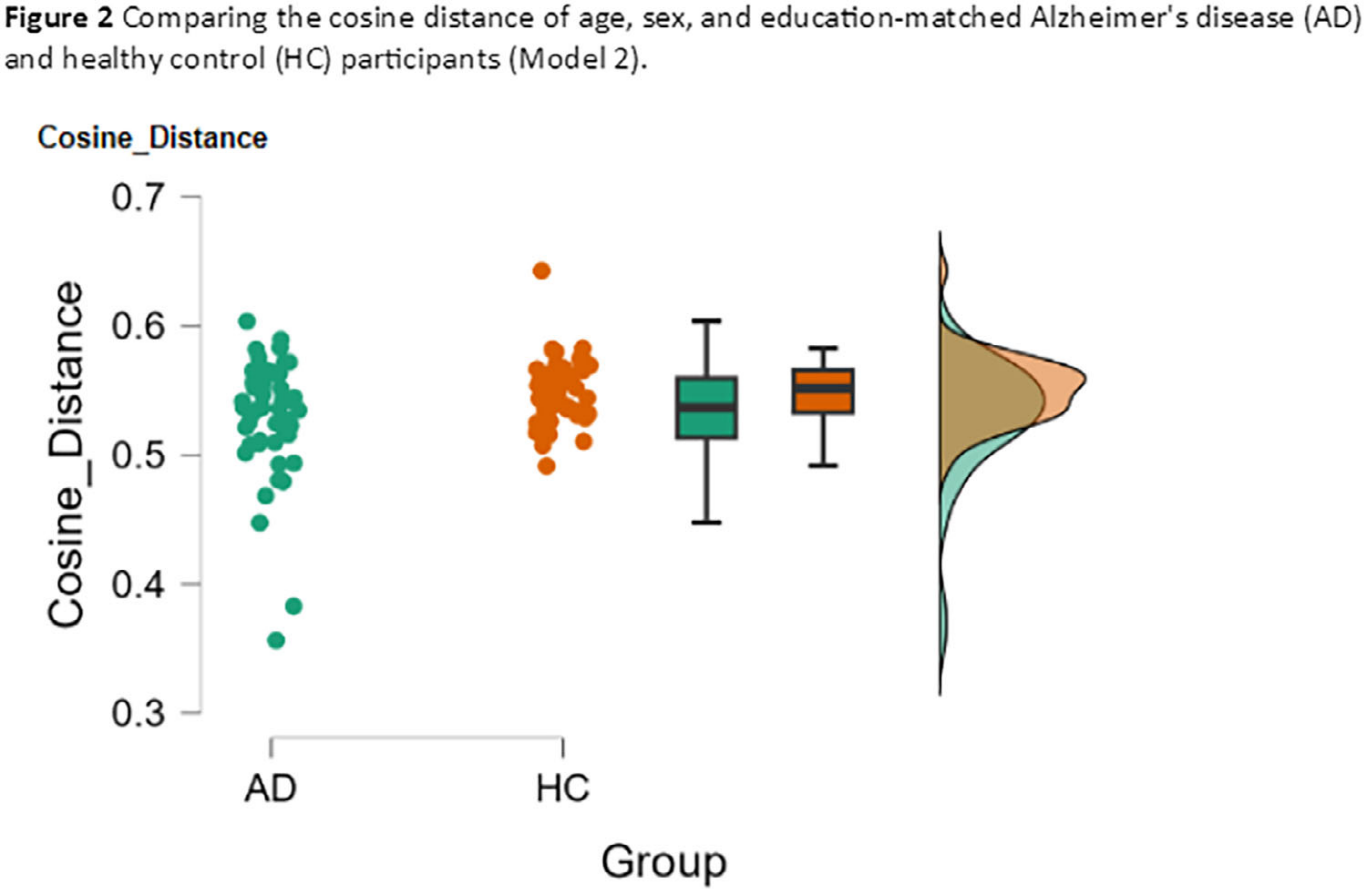# Semantic mapping in Alzheimer's disease: Measuring semantic distance using Natural Language Processing

**DOI:** 10.1002/alz70858_101145

**Published:** 2025-12-25

**Authors:** Sarah Pfeiffer, Esther Kim, Ikjyot Singh, Tanya Dash

**Affiliations:** ^1^ University of Alberta, Edmonton, AB, Canada

## Abstract

**Background:**

Semantic knowledge deficits are a common characteristic in people with dementia, yet how the brain maps words and meaning remains unclear. Semantic distance is a measure used to quantify the distance between two concepts in semantic space2. Through the use of verbal fluency data, we examined the semantic distance of animal names to gain insight into semantic mapping patterns. Our objective was to determine whether semantic distance can serve as a linguistic marker to identify differences in semantic representations between individuals with Alzheimer's disease (AD) and healthy controls.

**Method:**

Using verbal fluency data from DementiaBank1, Natural Language Processing (NLP) techniques were applied to generate the semantic distance for individual responses for all participants. We then compared the average semantic distance across groups using two models. Model 1: the AD group (*N* = 76) and the healthy control group (*N* = 76) were matched on age. Model 2: the AD group (*N* = 51) and healthy control group (*N* = 51) were matched for age, sex, and education. Analysis was completed using Python.

**Result:**

Results in Model 1 showed that the AD group had a significantly shorter semantic distance than the healthy control group, with a medium effect size. Findings in Model 2, which involved a more strict participant matching process, showed similar results to Model 1, with a small effect size.

**Conclusion:**

Semantic distance, as measured using NLP techniques, has the potential to distinguish individuals with Alzheimer's disease from those without. A shorter semantic distance, which was observed in the AD group, may reflect an inefficient and disrupted semantic network. An impaired semantic system may restrict access to semantic representations that are more distant from the core concept. Enhancing understanding of semantic systems in AD may lead to a more nuanced linguistic profile that can be used to monitor disease progress. This study contributes to the growing evidence for the role of computational tools in analyzing language‐based assessments to advance understanding of semantic deficits in dementia.

**References**

1. DementiaBank. https://dementia.talkbank.org/

2. Reilly, J., et al. (2024). https://doi.org/10.3758/s13423‐024‐02556‐7